# Proteomic analysis of tyrosine phosphorylation during human liver transplantation

**DOI:** 10.1186/1477-5956-5-1

**Published:** 2007-01-02

**Authors:** Anouk Emadali, Peter P Metrakos, Fariba Kalantari, Tarek Boutros, Daniel Boismenu, Eric Chevet

**Affiliations:** 1 Department of Surgery, McGill University, Montreal, Quebec, Canada; 2 Genome Québec Innovation Centre, McGill University, Montreal, Quebec, Canada; 3 Departement of Medecine, McGill University, Montreal, Quebec, Canada; 4 Department of Anatomy and Cell Biology, McGill University, Montreal, Quebec, Canada; 5 Team AVENIR, INSERM E362, Université Bordeaux 2, Bordeaux, France; 6 CEA/Grenoble, Grenoble, France

## Abstract

**Background:**

Ischemia-reperfusion (I/R) causes a dramatic reprogramming of cell metabolism during liver transplantation and can be linked to an alteration of the phosphorylation level of several cellular proteins. Over the past two decades, it became clear that tyrosine phosphorylation plays a pivotal role in a variety of important signalling pathways and was linked to a wide spectrum of diseases. Functional profiling of the tyrosine phosphoproteome during liver transplantation is therefore of great biological significance and is likely to lead to the identification of novel targets for drug discovery and provide a basis for novel therapeutic strategies.

**Results:**

Using liver biopsies collected during the early phases of organ procurement and transplantation, we aimed at characterizing the global patterns of tyrosine phosphorylation during hepatic I/R. A proteomic approach, based on the purification of tyrosine phosphorylated proteins followed by their identification using mass spectrometry, allowed us to identify Nck-1, a SH_2_/SH_3 _adaptor, as a potential regulator of I/R injury. Using immunoblot, cell fractionation and immunohistochemistry, we demonstrate that Nck-1 phosphorylation, expression and localization were affected in liver tissue upon I/R. In addition, mass spectrometry identification of Nck-1 binding partners during the course of the transplantation also suggested a dynamic interaction between Nck-1 and actin during I/R.

**Conclusion:**

Taken together, our data suggest that Nck-1 may play a role in I/R-induced actin reorganization, which was previously reported to be detrimental for the hepatocytes of the transplanted graft. Nck-1 could therefore represent a target of choice for the design of new organ preservation strategies, which could consequently help to reduce post-reperfusion liver damages and improve transplantation outcomes.

## Background

Protein phosphorylation is considered to be one of the major determinants regulating a large spectrum of biological processes [1]. It is a key reversible modification occurring mainly on serine, threonine and tyrosine residues, by acting as a switch to turn "on" or "off" a protein activity or a cellular pathway [2]. Although far less frequent than serine/threonine phosphorylation [3], tyrosine phosphorylation plays a key role in regulating many different processes in eukaryotic organisms, such as growth or cell cycle control, differentiation, cell shape and movement, gene transcription, synaptic transmission and insulin action [4]. Phosphotyrosine (PY) residues are recognized by specialized binding domains on other proteins such as Src Homology 2 (SH_2_), PY interaction domains (PID) or PY binding domains (PTB) [5], and such interactions are used to initiate and promote intracellular signalling. Tyrosine phosphorylation therefore plays a prominent role in signal transduction, but yet, these signalling pathways have been difficult to identify, in part because of their complexity and in part because of low cellular levels of tyrosine phosphorylation.

Recent advances, including the availability of the complete human genome sequence [6], have set the stage for comprehensive or global proteomic analyses. At the same time, mass spectrometry was emerging as a reliable and sensitive tool for protein identification and protein phosphorylation site determination [7] and now represents a method of choice for the large scale analysis of protein phosphorylation [3]. After affinity-based enrichment of tyrosine phosphorylated proteins using specific anti-PY antibodies, phosphorylation analysis by mass spectrometry is generally accomplished in a two-step process. Proteins of interest are proteolytically digested, usually with trypsin, and the resulting peptides are analyzed to determine those which are phosphorylated. Separation of tryptic peptides using liquid chromatography (LC) is an efficient strategy to decrease sample complexity. Subsequently, peptides are further analyzed by tandem mass spectrometry (MS/MS), i) to identify the corresponding proteins and ii) to determine the precise location of the phosphorylation site(s). Phosphopeptides can be identified simply by examination of the list of observed peptide masses for mass increases of 80 Da (the added mass of the phosphate group) compared with the list of expected peptide masses.

Ischemia/reperfusion (I/R) constitutes a major injury in a variety of circumstances including as myocardial infraction, cerebral ischemia, stroke, hemorrhagic shock and organ transplantation [8]. During liver transplantation, donor organs experience some degree of preservation injury which is a cold I/R injury. Indeed, cold storage of the organ slows down metabolic processes that may lead to cell death and organ failure during the ischemic phase. It represents therefore one of the most fundamental component of successful organ preservation during transplantation, but also leads to specific secondary damages [9] The magnitude of preservation injury is a critical determinant for the success of liver transplantation. However, despite an intense investigative effort, the global, multifactorial and complex cell response initiated upon ischemia and reperfusion remains unclear.

In an attempt to uncover novel aspects of this intricate response, we have used a proteomic approach to characterize the cellular pathways regulated upon I/R during human liver transplantation. This led us to identify the adaptor protein Nck-1 as a major PY-containing protein whose phosphorylation level is regulated upon I/R. Moreover, we show that Nck-1 tyrosine phosphorylation coincides with changes in its sub-cellular localization and association with actin cytoskeleton. Our data provide the first evidence for Nck-1 tyrosine phosphorylation upon I/R in human liver and suggest that this protein may represent an important player in hepatocytes stress response.

## Results

### Identification of tyrosine-phosphorylated proteins upon I/R

**Figure 1:**
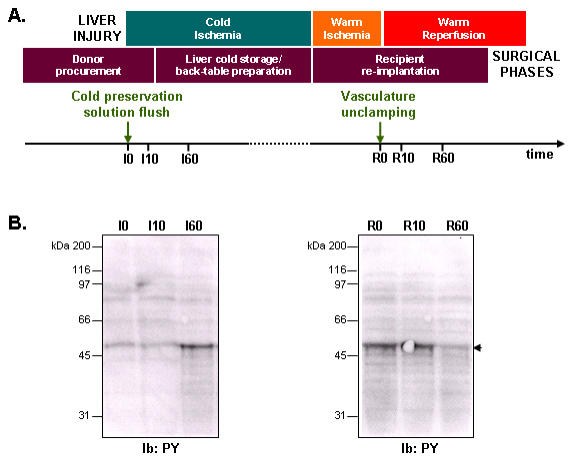
**Biopsy collection and evaluation of global tyrosine phosphorylation patterns upon I/*R***. **A**. Schematic representation of biopsy collection timing illustrating the surgical phases and corresponding liver injury. **B**. Representative immunoblot analysis of tyrosine phosphorylation on total fractions for 0, 10 and 60 min ischemia (I0, I10, I60) and 0, 10 and 60 min reperfusion (R0, R10, R60) liver protein extracts. N = 2 on 3 independent pools of 3 liver biopsy protein extracts.

**Figure 2:**
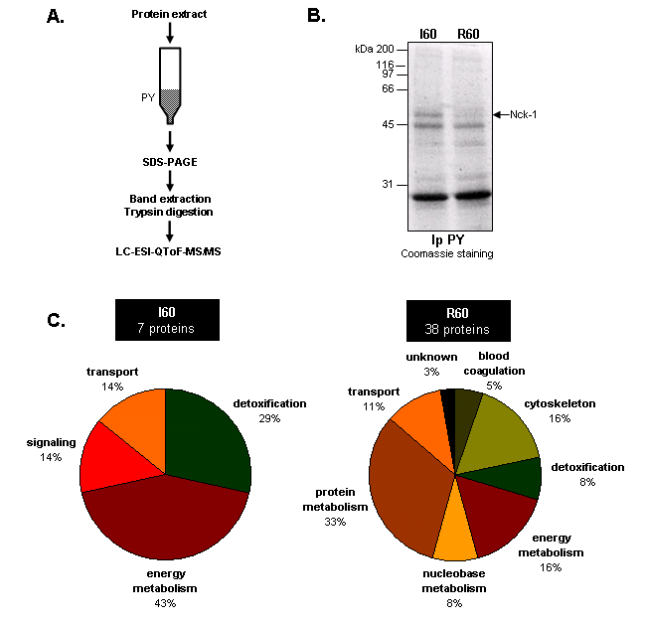
**Identification of tyrosine phosphorylated proteins upon I/R**. **A**. Schematic representation of the approach used for tyrosine phosphorylated proteins identification. PY matrix: anti-phosphotyrosine antibodies coupled to agarose beads. **B**. Representative SDS-PAGE experiment after tyrosine immunoprecipitation (Ip PY) of 60 min ischemia (I60) and 60 min reperfusion (R60) protein extracts. The band marked by an arrow has been further identified as the SH_2_/SH_3 _adaptor Nck-1. N = 1 on 2 independent pools of 3 liver biopsy protein extracts. **C**. Pie-chart representation of the total number of proteins identified with a significant Mascot score by at least one unique peptide for the 60 min ischemia (I60, left panel) and the 60 min reperfusion (R60, right panel). The proteins were classified in functional families according to their GO (Gene Ontology) annotation.

To better characterize the signalling pathways activated during human liver transplantation, we aimed to identify phosphotyrosine (PY) containing proteins whose phosphorylation was regulated upon I/R. To this end, we used liver biopsies collected as described in Figure 1A. Protein extracts (I0, I10, I60 corresponding to 0, 10 and 60 min ischemia and R0, R10, R60 corresponding to 0, 10 and 60 min reperfusion, respectively) were prepared as described in the Methods section and processed for immunoblot analyses using anti-PY antibodies. This allowed us to obtain a global tyrosine phosphorylation pattern during I/R (Figure 1B). Although, no significant change was observed for the large majority of the proteins protein detected, a major protein with a molecular weight of approximately 50 kDa was subjected to a dramatic change in its tyrosine phosphorylation status during both phases of the transplantation. An important tyrosine phosphorylation increase during the ischemic phase and a consecutive decrease during reperfusion were observed. In order to identify PY-containing proteins during the two phases of the transplantation, proteins extracts from biopsies collected at 60 min ischemia (I60) and 60 min reperfusion (R60) were chromatographed on anti-PY antibodies coupled to agarose beads as schematically represented in Figure 2A. Proteins were then eluted, resolved by 1D SDS-PAGE and stained using G250 Coomassie Blue (Figure 2B). Noteworthy, a major Coomassie Blue stained band in the I60 tyrosine phosphorylated fraction was no longer detectable in the R60 fraction, indicating that this ~50 kDa band may contain PY proteins specific to the ischemic phase. This protein may most likely correspond to the major protein detected in Figure 1B. Each band visualized on the gel was then excised, trypsin digested and the resulting peptides analyzed using Liquid Chromatography – ElectroSpray Ionization – quadrupole Time of Flight – tandem Mass Spectrometry (LC-ESI-QToF) MS/MS as described in the Methods section. The proteins identified are listed in Tables 1 (I60) and 2 (R60) and represented after classification into functional families according to their GO (Gene Ontology) annotation (Figure 2C). We identified only 7 proteins in the 60 min ischemia fraction (Table 1). Interestingly, the ~50 kDa band disappearing between I60 and R60 was reproducibly identified as the SH_2_/SH_3 _containing adaptor protein Nck-1 (Table 1). Besides Nck-1, which represented the only signalling component of this fraction, the 6 other proteins identified were composed of abundant proteins involved in liver metabolic functions (energy metabolism and detoxification) and of blood proteins involved in transport (Table 1 and Figure 1C, left panel). These 6 proteins were also found in the 60 min reperfusion fraction (Tables 1 and 2), suggesting that they may either represent contaminants or be constitutively tyrosine phosphorylated. Interestingly, a larger data set of 37 proteins was identified for the 60 min reperfusion time point (Table 2 and Figure 1C, right panel). In addition to hepatocytes to proteins involved in liver metabolism (energy metabolism: 16% and detoxification: 8%) and blood proteins (blood coagulation: 5%, transport: 11%), a large proportion (33%) of these proteins were involved in protein synthesis (mainly ribosomal proteins). We also identified structural components of the cytoskeleton (16%) and proteins involved in nucleobase metabolism (8%), as well as 1 unknown protein. Although the computational prediction of serine, threonine and tyrosine phosphorylation using the NetPhos software indicated potential tyrosine phosphorylation sites for the large majority of the proteins identified (Table 1 and 2), only 3 of these proteins had been previously reported as tyrosine phosphorylated: the alpha chain of tubulin, vimentin and the 60S ribosomal protein, L8 (Table 2).

**Table 1:** Anti-PY binding proteins – 60 min ischemia

**Protein name**	**Accession number^¶^**	**Mascot score**	**Sequence Coverage**	**Peptide number**	**Subellular Localization**	**Biological Function**	Reported PY sites*	**Predicted PY sites+**
*Serum albumin precursor*	*P02768*	*410*	*22%*	*10*	*secreted*	*transport*	*none*	*108, 164, 365, 476, 521*
*Carbamoyl-phosphate synthetase 1*	*Q5R206*	*298*	*4%*	*5*	*mitochondrion*	*detoxification*	*none*	*443, 634, 768, 852, 872, 957, 959, 1032, 1305, 1450*
*Dihydrolipoamide branched chain transacylase*	*P11182*	*196*	*14%*	*6*	*mitochondrion*	*energy metabolism*	*none*	*36, 85, 130*
Nck adaptor protein 1	P16333	164	11%	4	cytosol	signalling	none	13, 112, 268, 339
*Phosphoenolpyruvate carboxykinase*	*P35558*	*70*	*2%*	*1*	*mitochondrion*	*energy metabolism*	*none*	*165, 279, 595*
*Enoyl-CoA hydratase*	*P30084*	*62*	*2%*	*1*	*mitochondrion*	*energy metabolism*	*none*	*35*
*Alcohol dehydrogenase [NADP+]*	*P14550*	*47*	*4%*	*2*	*cytosol*	*detoxification*	*none*	*49, 139, 240, 324*

Proteins identified in the 60 min ischemia and the 60 min reperfusion fractions are represented in italic.

^¶ ^Database: SwissProt

*source: SwissProt and Human Protein Reference Database

^+ ^source: NetPhos 2.0

**Table 2:** Anti-PY binding proteins – 60 min reperfusion

**Protein name**	**Accession number^¶^**	**Mascot score**	**Sequence Coverage**	**Peptide number**	**Subellular Localization**	**Biological Function**	Reported PY sites*	**Predicted PY sites+**
Actin, cytoplasmic 2	P63261	664	42%	19	cytosol	cytoskeleton	none	108,164,365,476,521
*Carbamoyl-phosphate synthetase 1*	*Q5R206*	*538*	*9%*	*11*	*mitochondrion*	*detoxification*	*none*	*443,634,768,852,872,957, 959,1032,1305,1450*
60S ribosomal protein L7	P18124	495	45%	10	cytosol-nucleus	protein metabolism	none	51,155,159,182
*Serum albumin precursor*	*P02768*	*393*	*17%*	*8*	*secreted*	*transport*	*none*	*108,164,365,476,521*
*Alcohol dehydrogenase [NADP+]*	*P14550*	*387*	*21%*	*8*	*cytosol*	*detoxification*	*none*	*49,139,240,324*
Unknown (protein for MGC:22633)	BC022319^1^	375	21%	8	unknown	unknown	none	14,341
*Dihydrolipoamide branched chain transacylase*	*P11182*	*349*	*17%*	*8*	*mitochondrion*	*energy metabolism*	*none*	*36,85,130*
Myosin heavy chain	P13533	316	6%	7	cytosol	cytoskeleton	none	162,284,311,387,411,502, 1377,1462,1490,1854
Protein disulfide isomerase-related protein	Q14554	218	10%	3	ER	protein metabolism	none	113,222,242,245,357,364, 488
60S ribosomal protein L6	Q02878	217	16%	4	cytosol-nucleus	protein metabolism	none	68,72,239
60S ribosomal protein L13a	P40429	210	27%	6	cytosol-nucleus	protein metabolism	none	53,191
60S ribosomal protein L18	Q07020	175	24%	4	cytosol-nucleus	protein metabolism	none	23,165
60S ribosomal protein L5	P46777	143	12%	3	cytosol-nucleus	protein metabolism	none	29,30,43,48,144,182,218, 239, 252
*Enoyl-CoA hydratase*	*P30084*	*107*	*3%*	*2*	*mitochondrion*	*energy metabolism*	*none*	*35*
Very-long-chain acyl-CoA synthetase	O14975	102	4%	2	ER	detoxification	none	81, 117, 261, 413, 460, 553, 610
Ku antigen, 70 kD	NP001460^2^	102	4%	2	nucleus	nucleobase metabolism	none	8, 30, 103, 361, 534, 559
Alpha-s1-casein	P47710	102	10%	2	secreted	transport	none	none
60S ribosomal protein L8	P62917	99	9%	3	cytosol-nucleus	protein metabolism	132	none
40S ribosomal protein S4	P62701	94	4%	1	cytosol-nucleus	protein metabolism	none	53, 120
Tubulin beta-1 chain	Q9H4B7	92	8%	5	cytosol	cytoskeleton	none	55, 106, 222, 310
Complement C1q subcomponent subunit A	P02745	86	6%	2	secreted	blood coagulation	none	144
ATP-dependent RNA helicase A	Q08211	83	3%	2	cytosol	nucleobase metabolism	none	9,21,43,45,149,200,218,616,727748,1155,1167,1189,1194, 1234
Galactokinase	P51570	69	4%	1	cytosol	energy metabolism	none	47,318
40S ribosomal protein S2	P60866	69	5%	2	cytosol-nucleus	protein metabolism	none	none
Beta-casein	P05814	65	8%	2	secreted	transport	none	32,109
Phosphoenolpyruvate carboxykinase	P35558	64	2%	1	mitochondrion	energy metabolism	none	165,279,595
40S ribosomal protein S3	P23396	64	3%	1	cytosol-nucleus	protein metabolism	none	36,87,107, 67
Alpha-fetoprotein	P02771	62	3%	1	secreted	transport	none	60, 151, 164, 426, 505, 521
ATP synthase beta chain	P06576	50	3%	1	mitochondrion	energy metabolism	none	247, 418
Tubulin alpha-ubiquitous chain	P68363	48	2%	1	cytosol	cytoskeleton	272	210,224,399,432,451
Heterogeneous nuclear ribonucleoprotein AB	Q53F64	48	4%	1	cytosol-nucleus	protein metabolism	none	235,272,307
Vimentin	P08670	44	4%	2	cytosol	cytoskeleton	116	10,29,52,275,290
Histone H1.3	P16402	44	11%	1	nucleus	nucleobase metabolism	none	none
Fibrinogen gamma chain	P02679	42	3%	1	secreted	blood coagulation	none	27,140,288,300,444,448
Alpha-tropomyosin	P09493	39	1%	1	cytosol	cytoskeleton	none	60,162,214,221,261,267
ATP synthase alpha chain	P25705	39	2%	1	mitochondrion	energy metabolism	none	299,476
60S ribosomal protein L4	P36578	37	3%	1	cytosol-nucleus	protein metabolism	none	160

Proteins identified in the 60 min ischemia and the 60 min reperfusion fractions are represented in italic.

^¶ ^Database: SwissProt except ^1^EMBL and ^2^GenBank

*source: SwissProt and Human Protein Reference Database

^+ ^source: NetPhos 2.0

### Validation of Nck-1 tyrosine phosphorylation upon I/R

**Figure 3:**
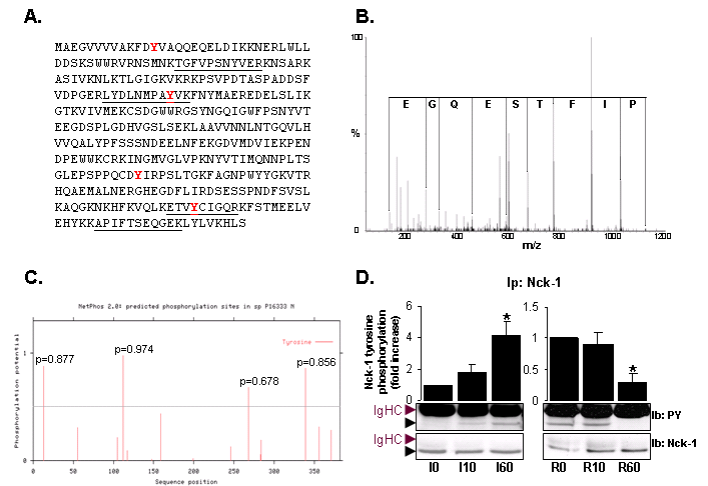
**Nck-1 tyrosine phosphorylation status upon I/R**. **A**. Nck-1 sequence: peptides identified by mass spectrometry are represented underlined, predicted tyrosine phosphorylation site are in red bold. **B**. Representative MS/MS spectrum corresponding to an Nck-1 specific peptide. **C**. Prediction of tyrosine-phosphorylation sites using the NetPhos software. **D**. Immunoblot analysis of Nck-1, using anti-Nck-1 antibody (Ib Nck-1) and Nck-1 tyrosine-phosphorylation status, using anti-phosphotyrosine antibody (Ib PY) following Nck-1 immunoprecipitation (Ip) on 60 min ischemia (I60) and 60 min reperfusion (R60) fractions. The band corresponding to Nck-1 is indicated by a black arrow. Immunoglobulins Heavy Chains (Ig HC) are indicated by a purple arrow. N = 2 on 3 independent pools of 3 liver biopsy protein extracts.

The SH_2_/SH_3 _containing adaptor Nck-1 has been linked to the regulation of multiple intracellular signal transduction events [10] with significant contribution to actin cytoskeleton remodelling. Consequently, Nck-1 could represent a critical target during I/R as actin cytoskeleton was previously reported to be subjected to major structural changes in livers subjected to I/R [11]. Four peptides were specifically attributed to Nck-1 (underlined on Nck-1 sequence, Figure 3A) in the 60 min ischemia fraction. The MS/MS spectrum corresponding to a peptide specific of Nck-1 is presented for reference (Figure 3B). Remarkably, besides the fact that the band corresponding to Nck-1 could not be detected by Coomassie Blue staining of the anti-PY purified fractions after 60 min of reperfusion (Figure 2B), Nck-1 was no longer identified in the anti-PY binding fraction after 60 min of reperfusion (Table 2), thus suggesting that Nck-1 may be either specifically tyrosine phosphorylated during the ischemic phase or associated to a phosphotyrosine-containing complex through its SH2 domain.

Nck-1 has previously been reported to be tyrosine phosphorylated in response to treatment with growth factors [12,13], but no information relative to the precise PY site is yet available in the literature. Noteworthy, the NetPhos software predicted four potential tyrosine phosphorylation sites within the Nck-1 sequence; two of them being located within MS identified non phosphorylated peptides (Figure 3C and red bold on Nck-1 sequence, Figure 3A). We experimentally confirmed that Nck-1 was indeed tyrosine phosphorylated by Nck-1 immunoprecipitation followed by immunoblot using anti-PY antibodies. A PY containing Nck-1 protein was detected as increasing during the first hour of ischemia, and decreasing during the first hour of reperfusion (Figure 3D, top panel). This was not due to major changes in the amount of Nck-1 protein immunoprecipitated as indicated by the blot using anti-Nck-1 antibodies (Figure 3D, bottom panel, and graph). This pattern further confirms that the ~50 kDa protein observed in Figure 1B may indeed represent Nck-1. This result also validates both Nck-1 tyrosine phosphorylation, as determined in our mass spectrometry analysis and the relevance of our approach to identify tyrosine phosphorylated proteins modulated during human liver transplantation.

### Nck-1 expression and sub-cellular localization in liver tissue upon I/R

**Figure 4:**
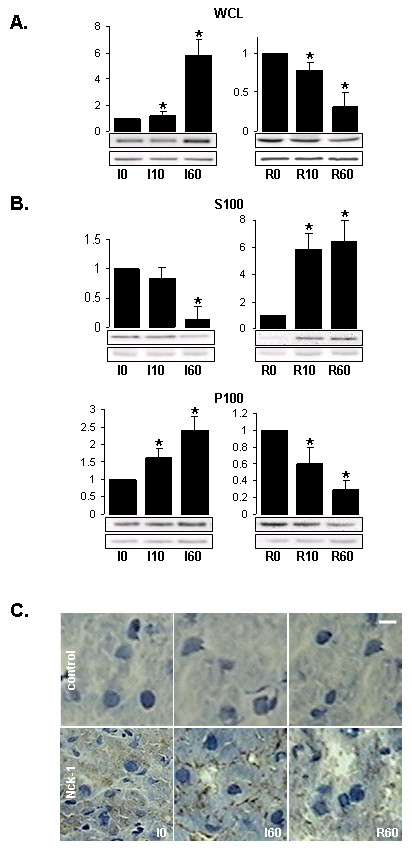
**Nck-1 expression and subcellular localization in human liver upon I/R**. **A**. Immunoblot analysis of Nck-1 from total fractions of 0, 10 and 60 min ischemia (I0, I10, I60) and 0, 10 and 60 min reperfusion (R0, R10, R60) liver protein extracts (WCL: Whole Cell Lysate) normalized to Intersectin (Int). N = 2 on 3 independent pools of 3 liver biopsy protein extracts. **B**. Immunoblot analysis of Nck-1 from insoluble (P100 – lower panel) and soluble (S100 – upper panel) fractions obtained after centrifugation of liver biopsies homogenized in the presence of 150 mM KCl respectively normalized to Ribophorin (Rib) and Intersectin (Int) for the 3 ischemia time points – 0 (I0), 10 (I10) and 60 (I60) min and the 3 reperfusion time points 0 (R0), 10 (R10) and 60 (R60) min. N = 2 on 3 independent pools of 3 liver biopsy protein extracts. **C**. Immunohistochemical detection of and Nck-1 on 8 μm liver tissue sections 0 and 60 min post-cold ischemia (I0 and I60) and 60 min post-reperfusion (R60). Cells were counterstained with haematoxylin (cells nuclei appear in blue). A representative experiment out of the 6 performed on independent ischemic and reperfused livers is shown. Scale bar = 10 μm.

To further characterize the functional relevance of Nck-1 tyrosine phosphorylation during human liver transplantation, we first assessed the expression levels of Nck-1 in the ischemic and reperfused livers using immunoblot analysis (Figure 4A). We observed an increased expression of Nck-1 during ischemia followed by a decrease during reperfusion. We then investigated the *in situ *distribution/localization of Nck-1 in liver during the different phases of the transplantation. Tissue biopsies were homogenized in the presence of 150 mM KCl. Particulate fractions were partitioned by centrifugation as described in the Methods section, and collected as P100 (insoluble pellet fraction) and S100 (soluble fraction). Interestingly, Nck-1 showed a decreased association with the insoluble components during the course of ischemia, whereas this pattern was reversed upon reperfusion (Figure 4B, upper panel). This observation correlates with the detection of increased amounts of this protein in the soluble fractions upon ischemia followed by a decrease upon reperfusion (Figure 4B, lower panel). These data suggest that Nck-1 is subjected to severe localization changes upon I/R in the liver. Immunohistochemical studies on liver sections upon I/R revealed that Nck-1 localized in hepatocytes' cytoplasm under basal conditions and concentrated at the cell periphery after 1 h of ischemia (Figure 4C, I60). This phenomenon appeared to be reversed upon reperfusion (Figure 4C, R60). Interestingly, Nck-1 followed the same redistribution that we previously observed for F-actin [11]. Since Nck-1 is known to link tyrosine phosphorylation induced by extracellular signals to downstream regulators of actin dynamics [14], this protein may therefore serve to promote actin reorganization at hepatocytes' periphery upon I/R.

### Determination of Nck-1 interacting partners upon I/R

**Figure 5:**
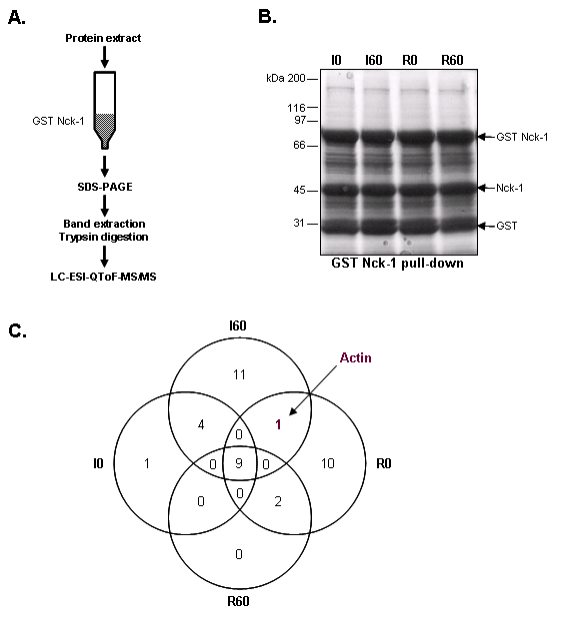
**Determination of Nck-1 interactants by GST pull-down**. **A**. Schematic representation of the approach used for tyrosine mass spectrometry identification of GST Nck-1 binding proteins. GST Nck-1 matrix: GST Nck-1 fusion protein coupled to sepharose beads. **B**. Representative SDS-PAGE experiment after GST Nck-1 pull-down of 0 (I0) and 60 min ischemia (I60) and 0 (R0) and 60 min reperfusion (R60) protein extract. Bands corresponding to GST Nck-1 fusion protein and cleavage products (Nck-1 and GST) are marked by arrows. N = 1 on 2 independent pools of 3 liver biopsy protein extracts. **C**. Venn diagram representation of the proteins identified after mass spectrometry analysis of GST Nck-1 binding proteins.

To better characterize the involvement of Nck-1 in actin remodelling in livers subjected to I/R, we aimed at identifying Nck-1 interaction partners during the course of transplantation. To this end, GST Nck-1 pull-downs were carried out using I0, I60, R0 and R60 protein extracts pre-cleared with GST (see Methods). Nck-1 binders were eluted, resolved by SDS-PAGE and the bands visualized on Coomassie Blue stained SDS-PAGE gels were processed for mass spectrometry analysis as represented in Figure 5A. No significant changes in proteins binding to GST Nck-1 could be detected by Coomassie Blue staining (Figure 5B). However, besides the bait proteins (Nck-1 and GST) and 9 proteins commonly identified for the 4 time points (Figure 4C), we also identified Nck-1 interacting proteins specific to I60 (11) or R0 (10) (see Additional File 1). Although using this approach, we did not find any of the proteins previously reported to interact directly with Nck-1, we identified actin as a major Nck-1 binding partner specific to the ischemic phase (after 60 min of ischemia -I60- and just before reperfusion -R0-, but not for the other time points). We believe that the absence of known Nck-1 interactants in our list may result from their generally low abundance which could then be masked by the presence of abundant liver metabolism proteins or by the fact that we are using an exogenous Nck-1 molecule instead of the endogenous as a trap. The presence of actin in association with Nck-1 during the ischemic phase, however, correlates with our immunohistochemistry data and, as a consequence, strengthens the hypothesis of Nck-1 involvement of in ischemia-induced actin dynamics.

## Discussion

Ischemia-reperfusion injury represents a major determinant for the success of liver transplantation. Such a complex and multifactorial cell response implies the concerted activation of major signalling cascades [15]. Subsequently, regulation of I/R injury must involve critical phosphorylation events, even though few of them have been characterized to date [11,16-18]. Protein tyrosine kinases and protein tyrosine phosphatases play a key role in cell signalling, and the recent successes of specific tyrosine kinase inhibitors in cancer treatment [19] strongly validate the clinical relevance of the research carried out on tyrosine phosphorylation in various patho-physiological contexts. Functional profiling of the tyrosine phosphoproteome during the course of liver transplantation should very likely lead to the identification of novel targets for drug discovery and provide the basis for novel molecular diagnostic approaches.

Analysis of the entire cellular phosphoprotein content, the so-called phosphoproteome, is an attractive study subject since technical advances of mass spectrometry methodologies can now lead to the accurate identification of post-translational modifications in a global and comprehensive manner [3]. However, a major obstacle in the study of phosphorylated proteins is that they comprise only a small fraction of the total protein contained in a cell lysate. Nevertheless, several studies based on anti-PY immunoprecipitation prior to mass spectrometry identification have been relatively effective at enriching and identifying even low-abundance tyrosine phosphorylated proteins [20-22].

Using such an approach, we identified 7 proteins for the 60 min ischemia and 37 proteins for the 60 min reperfusion protein extracts (Figure 2). The fact that far fewer proteins were identified after 60 min of ischemia can be explained by the depletion of cellular ATP at this stage [23]. This finding also correlates with our previously published studies as i) we recently showed that the number of proteins identified following IMAC-based enrichment was also lower during ischemia [11] and ii) using anti-phosphospecific antibodies, we demonstrated that for all the proteins tested, phosphorylation levels decreased upon ischemia and increased upon reperfusion [16]. This was indicative of the occurrence of events which could also be related to the depletion in high-energy phosphate carriers.

In our study, most of the proteins identified are involved in liver or blood basic metabolic functions. Consequently, they are unlikely to represent critical signalling components of I/R injury regulation but may rather indicate significant modulation of the metabolic rates of liver tissues upon transplantation. However, it is interesting to note the presence of several ribosomal proteins, whose involvement in protein translation could be linked to the post-reperfusion restoration of protein biosynthesis as we recently reported [16]. Moreover, the identification of two subunits of ATP synthase can be correlated to the re-establishment of energy stores [23].

As illustrated in previously reported studies, our approach did not lead to the identification of phosphorylation sites [22,24]. This may be due to the low representation/occurence of phosphotyrosine residues. Therefore, high levels of non-phosphorylated peptide may mask the detection of low-abundance phosphopeptides. In addition, the experimental conditions used for PY immunoprecipitation can lead to the identification of non-phosphorylated members of tyrosine phosphorylation-dependent protein complexes. Finally, the liver is especially rich in certain classes of metabolic enzymes, which, like for plasma proteomics [25] may mask the presence of less abundant signalling components. Recently, several teams partly overcame this problem by performing IMAC enrichment in phosphopeptides after phosphotyrosine containing proteins immunoprecipitation [26,27].

Although tyrosine phosphorylated protein identification by mass spectrometry represents a significant advance in the understanding of biological processes, we still believe that experimental validation of the tyrosine phosphorylation status remains a necessary step to confirm identifications. Noteworthy, this step can be easily achieved using specific anti-PY antibodies or in vitro phosphorylation techniques [3].

Here, we experimentally confirmed Nck-1 tyrosine phosphorylation during the ischemic phase of liver transplantation. In addition, we provide the first evidence that this SH_2_/SH_3 _adaptor, which physically bridges PY-containing regions to downstream components, can be itself tyrosine phosphorylated under real biological conditions. Then, we demonstrated that Nck-1 in vivo expression and subcellular localization are affected in liver tissue during I/R, correlating with its tyrosine phosphorylation status. We observed that Nck-1 follows the same I/R-dependent subcellular redistribution than that previously reported for actin and IQGAP1. The characterization of Nck-1 interacting partners also suggests an ischemia-induced association of Nck-1 with actin.

In many reports, Nck-1 has been associated to actin dynamics as a bridge between extracellular signals through cell surface receptors to actin nucleation via the WASP-Arp2/3 complex [28]. In views of our data and the literature, Nck-1 could therefore represent a critical regulator of ischemia-induced actin rearrangement at hepatocytes periphery. The fact that actin cytoskeleton remodelling and the related maintenance of hepatocytes canaliculi integrity were reported to have direct functional implications on liver transplantation outcome especially in term of i) bile secretion properties [29,30] and ii) cell's tolerance to I/R stress [11] also suggests that Nck-1 could represent a promising molecular target to reduce I/R-induced liver damages.

## Conclusion

In summary, this work consists in a proteomic analysis of human liver biopsies combined with the targeted enrichment of PY-containing proteins during different phases of the transplantation. This approach led to the identification of Nck-1. We confirm that Nck-1 is specifically tyrosine-phosphorylated during the ischemic phase and correlate this observation with change in expression and sub-cellular localization of this protein. Mass spectrometry identification of Nck-1 binding partners has allowed us to formulate the hypothesis that Nck-1 could be related to the ischemia-induced actin reorganization. The elucidation of this molecular target may therefore on a longer term allow the design of therapeutics improving/accelerating graft recovery and improving transplantation outcomes.

## Methods

### Tissue collection

0.5 cm^3 ^biopsies were surgically removed in the course of 34 liver transplantation procedures performed within McGill University Health Centre with approval of the institution's ethic committee (ERB 05-003) as previously described [11,16] and as schematically represented in Figure 1A. Briefly, ischemic liver biopsies were taken shortly before the donor liver clamping (I0), and 10 and 60 min (I10 and I60) after perfusion of the liver with cold organ preservation solution (Belzer^®^). Reperfused liver biopsies were collected during re-implantation in the recipient patient, just before clamp removal (R0), and respectively 10 and 60 minutes after the blood flow has been re-established in the hepatic portal vein (R10 and R60). The average cold ischemia phase was 8 h 30 ± 2 h 15 whereas the average warm ischemia time was 48 min ± 26 min.

### Protein extraction

Proteins were extracted from each tissue sample by homogenization and solubilization in phosphate buffer saline (PBS) added with 1% TX100 1 mM NaF, 1 mM Na_3_VO_4 _and protease inhibitors (Complete^®^, Roche Diagnostics, Laval, QC). Equal protein amounts from 3 independent ischemia or reperfusion liver biopsies were pooled for each time-point to reduce the variability between individual patients.

### Immunoprecipitation

Protein extracts (1 mg) were incubated overnight at 4°C with mouse monoclonal anti-phosphotyrosine antibodies covalently coupled to agarose beads (PY20, Sigma, St Louis, MO) or rabbit anti Nck-1 immune serum, kindly provided by Dr Louise Larose, McGill University, Montreal, QC [31]. Immune complexes were collected following incubation with protein G-Sepharose beads (GE HealthCare, Baie d'Urfé, QC) for 45 min at 4°C. After three to five washes in PBS, proteins were eluted using Laemmli sample buffer and resolved by 1D SDS-PAGE prior to mass spectrometry analysis or immunoblotting.

### Mass spectrometry

Following separation of the proteins purified as described above by 1D SDS-PAGE and Coomassie R-250 staining, each band was excised, transferred to a 96 wells tray, dehydrated with acetonitrile and washed by two cycles of 10 min in 100 mM (NH_4_)_2_CO_3 _before the addition of an equal volume of acetonitrile. The destained gel slices were then treated for 30 min with 10 mM dithiothreitol to reduce cystinyl residues and for 20 min with 55 mM iodoacetamide to effect alkylation. After an additional round of (NH_4_)_2_CO_3 _and acetonitrile washes, the slices were extracted with acetonitrile at 37°C. They were then incubated with trypsin (6 ng/μl in 50 mM (NH_4_)_2_CO_3_) for 5 h at 37°C and the peptides were extracted in 1% formic acid/2% acetonitrile followed by two further extractions with additions of acetonitrile. All treatments were performed robotically using a MassPrep Workstation (Micromass, Manchester, UK). All extracts from a given gel slice were combined in the corresponding well of another 96 wells tray. This tray was transferred to the autosampler of a CapLC (Waters, Milford, MA) for mass spectrometry analysis on a QTOF2 (Micromass, Manchester, UK) upgraded with EPCAS™ (Embedded PC Acquisition System). A volume of 20 μL of sample was injected at a flowrate of 30 μL/min with pump C to a μ-Precolumn™ (LC Packings, Amsterdam, NL) filled with C18 Pepmap 100 (300 μm ID × 5 mm, 5 μm, 100 Å) held on a 10 port Cheminert^® ^valve (VICI Valco Canada, Brockville, ON). After 5 min of washing, the 10 port valve was actuated so the acetonitrile gradient from pump A and B eluted the peptides toward the PicoFrit column (New Objective, Woburn, MA) filled with BioBasic^® ^C18 stationary phase (75 μm ID, 10 cm, 5 μm, 300 Å). Gradient solvent was delivered at a flowrate of 1 μL/min and was splitted to 200 nL/min for the PicoFrit™ column with a splitting tee. Solvent A was water (formic acid 0,1%) and solvent B was acetonitrile (formic acid 0,1%). The linear gradient was set from 5% B to 40% in 20 min, from 40% to 70% in 5 min, from 70% to 95% in 5 min, held at 95% for 7 min and brought back to 5% in 10 min. The PicoFrit™ column was installed on a nanospray probe so that the spraying tip was near the sampling cone of the mass spectrometer. Voltage on the capillary was adjusted to get a nice plume during elution of peptides. Acquisitions were done in Data Directed Acquisition mode (DDA) while a 1 second survey scan was first done from 350 to 1600 *m/z*. The four most intense doubly and triply charged ions were selected to undergo MS/MS fragmentation in 1 second scans from 50 to 2000 *m/z*. The collision energies were determined automatically by the instrument based on the *m/z *values and charged states of the selected peptides. MS/MS fragmentation stopped when the total ion current was lower than 3 counts or up to a maximum acquisition time of 5 seconds, whatever came first.

### Mass spectrometry data processing and analyses

The MS/MS data were peaklisted (ProteinLynx, Micromass, UK) and submitted to a local Mascot database search software (Matrix Science, UK) for search analysis against the NCBI mammalian non-redundant database with a confidence level of 95% or greater. Specific and shared peptides with an equal or greater score than the identity score were kept and recorded for each band. Proteins for which at least one specific peptide (i.e. peptide found specifically in their cognate protein) was identified with a significant Mascot score in the two replicates were retained.

### Cell fractionation and immunoblotting

Proteins were extracted from each tissue sample by homogenization in 150 mM KCl, 10 mM Tris-HCl, pH 7.5, 2.5 mM MgOAc, 4 mM imidazole pH 7.4 added with 1 mM NaF, 1 mM Na_3_VO_4 _and protease inhibitors (Complete^®^, Roche Diagnostics, Laval, QC) and pre-cleared. Insoluble fractions (pellet – P100) were then partitioned from soluble fraction (supernatant – S100) following centrifugation at 100,000 rpm for 30 min in a TLA-100.2 rotor (Beckman Coulter, Fullerton, CA). Immunoblot analyses were carried out as described previously [32].

### Immunohistochemistry

Liver tissues sections (8 μm thick) on glass slides were fixed in 3.7% formaldehyde. Tissue sections were peroxidase-immunostained with Nck-1 antibody using the Envision+^® ^rabbit kit (Dako, Mississauga, ON) according to the manufacturer's instructions. Sections were then counterstained using hematoxylin (Vector, Burlington, ON) then dehydrated and mounted using Permount (Fisher, Nepean, ON).

### Nck-1 GST pull-down

BL21 bacteria expressing pGEX-2TK Nck-1 GST [33] were sonicated 3 × 30 s on ice and lysed in PBS added with 1% TX100, 1 mM NaF, 1 mM Na_3_VO_4 _and protease inhibitors (Complete^®^, Roche Diagnostics, Laval, QC) for 30 min on ice. After 30 min centrifugation at 4°C, fusion proteins were purified with glutathione-Sepharose beads (GE HealthCare, Baie d'Urfé, QC) according to manufacturer instructions. Beads were then incubated with 1 mg of protein extract pre-cleared with GST as described previously [34], collected, washed three times with PBS and resuspended in Laemmli sample buffer prior protein separation on 1D SDS-PAGE and mass spectrometry analysis.

### Statistical evaluation

Immunoblots analyses were performed on 3 pools of three livers (9 independent livers) and in two replicates. Immunostainings were carried out on 6 independent livers and mass spectrometry analyses were performed on 2 pools of three livers (6 independent livers). Immunoblot data were analysed and quantified using the FluorChem software (Alpha Innotech, San Leandro, CA). Data are expressed as means ± SD (Standard Deviation). Comparisons between the 0 time point (control) and the 10 and 60 min time point (treatment) were performed using a Wilcoxon signed rank test. A value of p < 0.05 was considered as significant and marked with an asterisk.

## Abbreviations

1D SDS-PAGE: One-dimension Sodium Dodecyl Sulfate-Polyacrylamide Gel Electrophoresis

ESI: ElectroSpray Ionization

GST: Glutathione S-Transferase

I/R: Ischemia/Reperfusion

kDa: kiloDaltons

LC: Liquid Chromatography

qToF: quadrupole Time of Flight

MS: Mass Spectrometry

MS/MS: tandem Mass Spectrometry

PBS: Phosphate Buffered Saline

PY: Phospho-tYrosine

SD: Standard Deviation

SH_2_: Src Homology 2

SH_3_: Src Homology 3

## Competing interests

The author(s) declare that they have no competing interest.

## Authors' contributions

AE participated to study conception, performed the experimental part (except for mass spectrometry data acquisition), analyzed the data and drafted the manuscript.

PPM provided access to clinical samples.

FK performed immunofluorescence analyses

TB realized tissue sections

DB performed mass spectrometry data acquisition.

EC conceived the study, participated in its design and coordination and revised the manuscript.

All authors have read and approved the manuscript.

## Supplementary Material

Additional File 1 **Nck-1 interacting partners during I/R**. This table lists the proteins identified using mass spectrometry after GST Nck-1 pull down. The proteins identified for all time points are underlined in grey. Click here for file
